# *Pfmdr1 *copy number and arteminisin derivatives combination therapy failure in falciparum malaria in Cambodia

**DOI:** 10.1186/1475-2875-8-11

**Published:** 2009-01-12

**Authors:** Pharath Lim, Alisa P Alker, Nimol Khim, Naman K Shah, Sandra Incardona, Socheat Doung, Poravuth Yi, Denis Mey Bouth, Christiane Bouchier, Odile Mercereau Puijalon, Steven R Meshnick, Chansuda Wongsrichanalai, Thierry Fandeur, Jacques Le Bras, Pascal Ringwald, Frédéric Ariey

**Affiliations:** 1 Institut Pasteur in Cambodia, 5 Monivong Boulevard, P.O Box 983, Phnom Penh, Cambodia; 2 Department of Epidemiology, UNC School of Public Health, Chapel Hill, NC, USA; 3 National Center for Parasitology, Entomology and Malaria Control (CNM), Phnom Penh, Cambodia; 4 World Health Organization, Phnom Penh, Cambodia; 5 Pasteur Genopole Ile de France, Institut Pasteur, France; 6 Institut Pasteur, Unité d'Immunologie Moléculaire des Parasites, CNRS URA 2581, France; 7 Independent Scholar, 130 Sub Street, Bangkok 10500, Thailand; 8 Faculté des sciences pharmaceutiques et biologiques, EA209 Université Paris Descartes, France; 9 Global Malaria Department, World Health Organization, Headquarters, Geneva, Switzerland

## Abstract

**Background:**

The combination of artesunate and mefloquine was introduced as the national first-line treatment for *Plasmodium falciparum *malaria in Cambodia in 2000. However, recent clinical trials performed at the Thai-Cambodian border have pointed to the declining efficacy of both artesunate-mefloquine and artemether-lumefantrine. Since *pfmdr1 *modulates susceptibility to mefloquine and artemisinin derivatives, the aim of this study was to assess the link between *pfmdr1 *copy number, in vitro susceptibility to individual drugs and treatment failure to combination therapy.

**Methods:**

Blood samples were collected from *P. falciparum*-infected patients enrolled in two in vivo efficacy studies in north-western Cambodia: 135 patients were treated with artemether-lumefantrine (AL group) in Sampovloun in 2002 and 2003, and 140 patients with artesunate-mefloquine (AM group) in Sampovloun and Veal Veng in 2003 and 2004. At enrollment, the in vitro IC_50 _was tested and the strains were genotyped for *pfmdr1 *copy number by real-time PCR.

**Results:**

The *pfmdr1 *copy number was analysed for 115 isolates in the AM group, and for 109 isolates in the AL group. Parasites with increased *pfmdr1 *copy number had significantly reduced in vitro susceptibility to mefloquine, lumefantrine and artesunate. There was no association between *pfmdr1 *polymorphisms and in vitro susceptibilities. In the patients treated with AM, the mean *pfmdr1*copy number was lower in subjects with adequate clinical and parasitological response compared to those who experienced late treatment failure (n = 112, p < 0.001). This was not observed in the patients treated with AL (n = 96, *p *= 0.364). The presence of three or more copies of *pfmdr1 *were associated with recrudescence in artesunate-mefloquine treated patients (hazard ratio (HR) = 7.80 [95%CI: 2.09–29.10], N = 115), *p *= 0.002) but not with recrudescence in artemether-lumefantrine treated patients (HR = 1.03 [95%CI: 0.24–4.44], N = 109, *p *= 0.969).

**Conclusion:**

This study shows that *pfmdr1 *copy number is a molecular marker of AM treatment failure in *falciparum *malaria on the Thai-Cambodian border. However, while it is associated with increased IC_50 _for lumefantrine, *pfmdr1 *copy number is not associated with AL treatment failure in the area, suggesting involvement of other molecular mechanisms in AL treatment failures in Cambodia.

## Background

Since the report of chloroquine resistance along the Thai-Cambodian border in the late 1950s [1], Southeast Asia has been an important focus for the emergence and spread of drug resistance in *Plasmodium falciparum*. In Cambodia, chloroquine resistance prompted the change to sulphadoxine-pyrimethamine (SP) as first-line treatment in the 1970s. Resistance to SP occurred and the first-line treatment was then changed to mefloquine monotherapy in 1993. However, by the mid-1990s, resistance to mefloquine exceeded 25% (RI-RIII) in western Cambodia [2]. After several clinical trials, the combination of artesunate with mefloquine (AM) was introduced in 2000 in the most multidrug-resistant areas in the west of the country [3]. This regimen initially had a 28-day parasitological cure rate of approximately 95%. After four years of monitoring the efficacy of AM for the treatment of uncomplicated falciparum malaria, there is now evidence that the in vitro susceptibility of Cambodian strains to mefloquine is decreasing [2,4]. There is a risk that, like resistance to chloroquine and SP, mefloquine resistance will spread beyond Southeast Asia.

Recent studies showed that increased copy number of *pfmdr1 *was the best overall predictor of mefloquine treatment failure [5-7]. Increased *pfmdr1 *copy number predicted failure even after chemotherapy with the highly effective mefloquine and artesunate combination [5]. Monitoring *pfmdr1 *copy number may be an effective surveillance tool for drug resistance in Southeast Asia [7].

Investigation of *pfmdr1 *polymorphisms and gene copy number were performed using isolates from western Cambodia, an area endemic for multidrug-resistant *falciparum *malaria. The data was then compared to the clinical as well as in vitro response outcome for two separate treatment groups: AM (combination of artesunate and mefloquine) and AL (combination of artemether and lumefantrine (Coartem^®^). The aim of this study was to determine whether *pfmdr1 *polymorphism and/or increased gene copy number was predictive of arylaminoalcohol (mefloquine and lumefantrine) and endoperoxide (artesunate and artemether) resistance in Cambodia.

## Methods

### Clinical study

The National Malaria Control Programme of Cambodia (CNM) conducted three therapeutic efficacy studies at Sampovloun Referral Hospital, Battambang Province (western Cambodia): 1. artemether-lumefantrine between June and August 2002 (AL2002, N = 55), 2. artemether-lumefantrine between October 2003 and March 2004 (AL2003, N = 80) [8] and 3. artesunate-mefloquine between October 2003 and March 2004 (AM2003, N = 55) [9]. One therapeutic efficacy study of AM was conducted at Veal Veng Referral Hospital, Pursat Province (western Cambodia) between July and August 2004 (AM2004, N = 85) [9]. The studies followed the WHO standard protocol for the assessment of the efficacy of anti-malarial drugs [10]. Briefly, all patients over six years of age and weight > 16 kg presenting with fever, defined as axillary temperature ≥ 37.5°C or history of fever during 24 h prior to consultation, and a positive smear with *Plasmodium falciparum *mono-infection, with parasite density of 1,000–150,000 parasites/μl, were included in the studies. Informed consent was given by the patient or by a parent/guardian for children. Enrolled patients had to stay in hospital until blood smear became negative and had to commit to completing the 28-day follow-up. Exclusion criteria are: one or more of the general danger signs or any sign of severe and complicated malaria, pregnancy, febrile diseases other than malaria and severe malnutrition, and known hypersensitivity or contraindication to the study drugs. A total dose of 12 mg/kg artesunate and 25 mg/kg mefloquine were given over 3 days (AM group). The daily 4 mg/kg of artesunate were divided into two equal doses, one in the morning and one in the evening on day 0, and 4 mg/kg single dose on day 1 and 2 with a maximum adult dose of 600 mg in total. The 25 mg/kg of mefloquine were also divided into two equal doses in the morning and in the evening on day 0. The maximum dose of mefloquine was limited to 1,500 mg for adults as the side effects are common beyond this dose [9]). In the artemether-lumefantrine study group (AL group), subjects were treated with 20 mg/kg artemether and 120 mg/kg lumefantrine the first day at 0 and 8 hours and twice daily (morning and late afternoon) on the following two days (Coartem^®^, Novartis, Switzerland), i.e. 8–12 h apart (the total dose of 24 tablets for an adult as recommended by the manufacturer). In the AL2003 study, each dose of artemether-lumefantrine was provided with 250 ml milk and 5 pieces of coconut biscuit [8]. Patients were checked by malaria smear for the presence of parasites on study days 1, 2, 3, 7, 14, 21 and 28. If fever occurred at any time between the scheduled study days, a malaria smear was also done. People who did not present on their own were actively sought for. The therapeutic response was classified as early treatment failure (ETF), late clinical failure (LCF), late parasitological failure (LPF) and adequate clinical and parasitological response (ACPR) according to WHO protocols.

### Site and blood sampling

Blood samples (5 ml) from patients were collected before treatment in EDTA Vacutainers, and were transported to the Institut Pasteur in Cambodia at 4°C within 48 hours of collection for in vitro assays. An aliquot of each isolate was frozen at -80°C for molecular analysis. If parasitaemia recurred, an additional blood sample was collected onto 3 M Whatman filter paper and transported to the laboratory at room temperature, then kept at -20°C until DNA extraction.

### Preparation of drugs and plates

Quinine hydrochloride was obtained from Sigma (Germany). Mefloquine, chloroquine diphosphate, artemether and artesunate were obtained from WHO/TDR Drug Discovery Research (Geneva). Lumefantrine was obtained from Novartis Pharma (Vietnam). Stock solutions of chloroquine diphosphate were prepared in water (Biosedra, France) and stock solutions of quinine hydrochloride, mefloquine, lumefantrine, artemether and artesunate were prepared in ethanol. Further two-fold serial dilutions were prepared in distilled water (Biosedra, France). The final concentrations ranged from 0.05 to 51.2 nM for artesunate, 1 to 1024 nM for mefloquine, 5 to 5120 nM for chloroquine, 6.2 to 6400 nM for quinine, 0.1953 to 200 nM for lumefantrine and 0.097 to 100 nM for artemether. Each concentration was used to coat two wells of a Falcon 96-well, flat-bottom plate (ATGC, France). Forty microliters of drug solutions prepared as described above was added to each well and plates were then dried at room temperature in a sterile cabinet. The pre-dosed plates were finally kept frozen at -20°C until use. Their suitability for in vitro testing was regularly monitored using reference strains maintained in continuous culture (at Institut Pasteur in Cambodia) and presenting known responses to the various drugs tested.

### In vitro assay

The in vitro drug sensitivity of the Cambodian isolates was assessed by use of a classical isotopic 48 h test [11]. Briefly, fresh blood samples were washed three times with RPMI 1640 medium (GibcoTM, Invitrogen Corporation, France) by centrifugation (800 *g*, 10 min). The parasites were then tested directly without culture adaptation. The infected erythrocytes (1.5% haematocrit, 0.1–1% parasitaemia) were suspended in complete RPMI medium supplemented with 10% AB+ human serum that was heat inactivated for 30 min at 56°C (Biomedia, France) and buffered with 25 mM/l Hepes and 25 mM/l NaHCO3. The mixture was distributed (200 μl per well) into the 96-well test plates that had been pre-coated with anti-malarial agents. Each plate included two drug-free control wells and one control well without parasites. The culture plates were incubated for 48 h at 37°C in a 5%CO2 atmosphere. [^3^H]-hypoxanthine (0.5 μCi/well; Amersham Biosciences, France) was used to assess parasite growth. Each isolate was tested once in duplicate in the microplates with serial dilutions of drugs. Drug response was quantified by monitoring [^3^H]hypoxanthine uptake in a Wallac MicroBeta Trilux counter (Perkin-Elmer, France). At the end of the incubation period, the plates were frozen at -20°C and thawed to lyse the cells. After collection on glassfiber filter paper using a cell harvester, the amount of [^3^H]-hypoxanthine incorporated into the parasites nucleoprotein was determined. The results of in vitro assay are expressed as the 50% inhibitory concentration (IC_50_), defined as the concentration at which 50% of the incorporation of [^3^H]-hypoxanthine was inhibited, as compared with in the drug-free control wells. Parasite growth was measured by using a log probit approximation to determine the IC_50_values.

### DNA extraction

Parasite DNA was extracted from frozen blood aliquots (200 μl) using High Pure PCR template preparation kit (Roche^®^) following the manufacturers protocol. Blood stored on filter paper was extracted using QIAamp DNA Mini Kit (Qiagen) [12].

### Genetic polymorphism

Paired samples taken at enrollment and at recurrence of parasitaemia were used to distinguish between recrudescence and re-infection. The number of variants in three polymorphic genes (*msp1, msp2 and glurp*) was determined as described previously [13,14]. If the sample taken at recurrence contained either a subset of, or the same variants as the enrollment specimen, the infection was classified as recrudescence. If not, the infection was classified as a re-infection. The amplification of a single fragment at these three loci indicated that the parasite population was mono-infected (single genotype). Detection of two or more PCR bands, at one or more loci, indicated that the isolate contained multiple infections.

PCR amplification and direct sequencing were used to determine the single nucleotide polymorphism (SNP) at four *pfmdr1 *sites (positions 86, 184, 1042, and 1246) at enrollment date. The primer sequences used for the analysis of the N86Y and Y184F SNPs were: first amplification, 5'-TTA AAT GTT TAC CTG CAC AAC ATA GAA AAT T-3' (forward) and 5'-CTC CAC AAT AAC TTG CAA CAG TTC TTA-3' (reverse); nested amplification, 5'-TGT ATG TGC TGT ATT ATC AG GA-3' (forward) and 5'-CTC TTC TAT AAT GGA CAT GGT A-3' (reverse). The primer sequences used for the analysis of the N1042D and D1246Y SNPs were: first amplification, 5'-AAT TTG ATA GAA AAA GCT ATT GAT TAT AA-3' (forward) and 5'-TAT TTG GTA ATG ATT CGA TAA ATT CAT C-3' (reverse); nested amplification, 5'-GAA TTA TTG TAA ATG CAG CTT TA-3' (forward) and 5'-GCA GCA AAC TTA CTA ACA CG-3' (reverse). PCR products were sent to Genopole, Institut Pasteur Paris (IPP) for sequencing. Sequencing reactions were performed from both ends using ABI Prism BigDye Terminator Cycle Sequencing-Ready Reaction kits, and were run on a 3700 Genetic Analyzer (Applied Biosystems) as described previously [15]. Sequencing results were assembled, individually visually examined, and aligned using ABI Prism^® ^SeqScape^® ^software version 2.5 (Applied Biosystems).

### Real time PCR

Real time PCR was performed with an ABI PRISM 7000 sequence detection system (Applied Biosystems, Foster City, CA). *Pfmdr1 *copy number was assessed for all enrollment samples using a previous assay [5]. This assay consisted of combining both the primers and a FAM-TAMRA probe specific to a conserved region of *pfmdr1*, and the primers and a VIC-TAMRA probe specific for *β-tubulin*, all in one reaction. The primers were synthesized by Sigma-Proligo (Proligo Singapore Pty Ltd) and the probes were synthesized by Applied Biosystems UK. All samples were run in duplicate in 25 μl reactions. The reagents used for each unknown sample or standard were 1X Abgene QPCR ROX Master mix (2X, Abgene), 300 nM of each forward and reverse *pfmdr1 *primer, 150 nM of *pfmdr1 *probe, 100 nM of each forward and reverse *β-tubulin *primer and *β-tubulin *probe, 2 μl of template DNA and sterile water (Biosedra, Fresenius Kabi, France). DNA from strains 3D7 and Dd2 were included on each plate. The 3D7 DNA and Dd2 DNA were acquired from MR4 [16]. The reaction mixtures were prepared at 4°C in a 96-well optical reaction plate (Applied Biosystems) covered with optical adhesive covers (Applied Biosystems). The thermal cycling conditions were 50°C for 2 min, 95°C for 15 min and then 50 cycles of 95°C for 15 s and 60°C for 1 min. The threshold was placed to optimize the threshold cycle (*C*_*T*_) value for the first standardization reaction, and all subsequent *C*_*T *_values were obtained by using the same threshold value. *Pfmdr1 *copy number was calculated according to the following formula: copy number = (E_*βtubulin*_)^CT(*βtubulin*)^/(E_*pfmdr*1_)^CT(*pfmdr*1)^. The Efficiency (E) of the *β-tubulin *was assumed to be 2. Each plate included 1 well of 3D7 DNA (which has a copy number of 1) and 1 well Dd2 (which has 4 copies). The efficiency of the *pfmdr1 *was back calculated using CT's for 3D7 and Dd2 in the previous formula [17,18].

### Statistical analysis

All statistical analyses were performed using STATA (StataSE 8, Stata Corporation, College Station, Texas). The in vitro activity of anti-malarials is expressed as the geometric means of the IC_50_s for all isolates. Drug concentrations were transformed into logarithms. Linear regression analysis was used to assess the relationship between the prevalence of parasite and *pfmdr1 *copy number. The association between *pfmdr1 *copy number and treatment failure or IC_50 _of each anti-malarial drug was tested by Wilcoxon rank-sum (Mann-Whitney) test. The relationship between molecular changes in *pfmdr1 *and treatment failure was estimated using survival analysis. Patients who had been lost to follow-up or who presented again with re-infection were included but censored at their last visit or when they were diagnosed with a re-infection. All variables were assessed for the proportional hazard assumption. The main outcome was time to recrudescence. Previously identified risks for treatment failure with mefloquine and artesunate, and with lumefantrine and artemether, were included in Cox's regression analysis when assessing the association of *pfmdr1 *polymorphisms and treatment response. For all statistical tests, the significance level was set at p = 0.05.

### Ethical approval

This study was approved by the National Ethics Committee for Health Research, Ministry of Health, Cambodia, and the Technical Review Group of WHO/WPRO.

## Results

### Genetic diversity

At enrollment, 187 of 227 (82%) patient blood samples contained only one parasite population, with a single genotype at the *msp1, msp2 *and *glurp *loci, whereas 40 (18%) had multiple infections containing two (38/227 or 17%) or three (2/227 or 1%) distinct genotypes. The number of parasite populations in three isolates remained undetermined due to the poor quality of typing with three markers. The mean number of clones per isolate was 1.18 and standard deviation was 0.41.

### *Pfmdr1 *polymorphism

**Figure 1:**
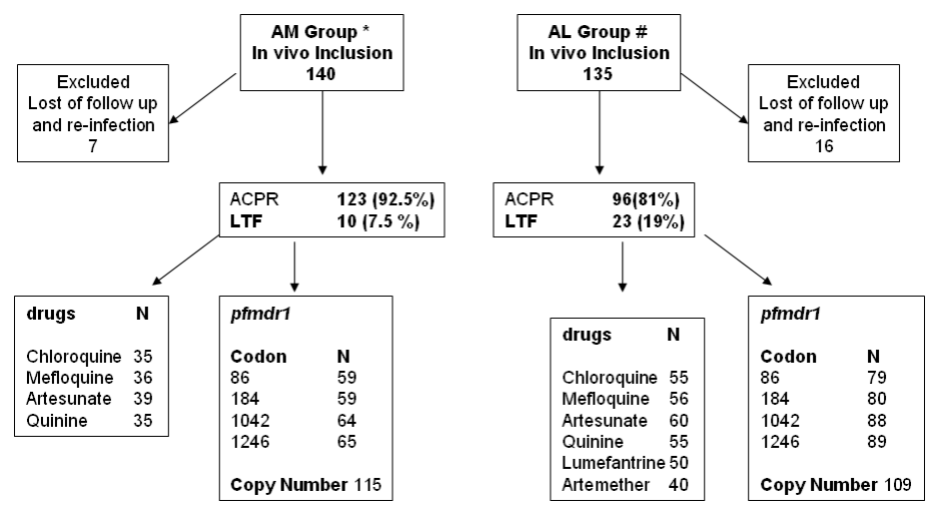
**Number of specimens analyzed for in vitro drug sensitivity, *pfmdr1 *polymorphism and *pfmdr1 *copy number according to the treatment group**. N: Number. ^#^Mey Bouth Denis et al, December 2006, Tropical Medicine and International Health [8]. *Mey Bouth Denis et al, September 2006, Tropical Medicine and International Health [9].

One hundred and sixty two patient blood samples were randomly selected for *pfmdr1 *sequencing at position of *pfmdr1*-86, 184, 1042 and 1246. One hundred and fifty six patient blood samples (96.3%) gave interpretable results (Figure 1). Out of these, 18 were unsuccessfully sequenced at *pfmdr1*-86, 17 were unsuccessful at *pfmdr1*-184, 1 was unsuccessful at *pfmdr1*-1042 and 1 was unsuccessful at *pfmdr1*-1246. No *Pfmdr1*-86-Tyr and *pfmdr1*-1246-Tyr mutants were observed. The results showed 125 (91%) *pfmdr1*-184-Phe mutant isolates and 11 (8%) *pfmdr1*-1042-Asp mutant isolates. No *Pfmdr1*-86-Tyr and *pfmdr1*-1246-Tyr mutants were observed.

### *Pfmdr1 *copy number

The *pfmdr1 *copy number was determined for 115 isolates in the AM group and for 109 isolates in the AL group, all collected at the day of enrollment (Figure 1). An overall mean copy number of 1.55 with a range from 0.56 to 4.91. Control DNA gave reproducible results as Dd2 had a mean copy number of 4.05 with a standard deviation of 0.47, and 3D7 control group had a mean copy number of 1.16 with a standard deviation of 0.14.

When rounded to the nearest integer, 182 (81%) isolates had a *pfmdr1 *copy number of one, 28 (12%) had two copies, 8 (4%) had three copies, and 6 (3%) had four copies. Overall, copy number was not related to baseline parasitaemia, age or gender (results not shown). No *pfmdr1 *polymorphisms were found to be associated with increased copy number. Genetic polymorphisms at the *msp1, msp2 *and *glurp *loci showed no indication of clonal population structure in high copy number isolates.

### In vitro drug sensitivity

In vitro sensitivities to artesunate, mefloquine, quinine and chloroquine were determined for 226 isolates. Microscopic examination showed that the parasite loads were between 0.02 and 14.4%, with a mean parasitaemia of 1.27%, CI 95% (1.02 – 1.52). Consistent with previous findings indicating that parasitaemia is critical for adequate in vitro testing, interpretable data for at least one of the drugs tested were obtained only for about half of the isolates (43.8%) (Figure 1). Other samples with low parasite counts (< 0.2%, n = 60) resulted in poor quality data and were not included in the study. In vitro sensitivity for artemether and lumefantrine were only tested on the AL group with 40 and 50 interpretable results, respectively (Figure 1). The geometric mean IC_50 _values for mefloquine, artesunate, quinine, chloroquine, lumefantrine and for artemether were 20.2 nM/l (n = 92, CI95% 16.1 – 25.3 nM, range 2.3 – 132.9), 1.3 nM/l (n = 99, CI95% 1.1 – 1.5 nM, range 0.3 – 11.1), 131.6 nM/l (n = 90, CI95% 115 – 150.7 nM, range 19.94 – 615.9), 196.1 nM/l (n = 90, CI95% 176.1 – 218.3 nM, range 54.1 – 663.3), 19.27 nM/l (n = 50, CI95% 15.8 – 23.4 nM, range 3.6 – 79.2) and 1.00 nM/l (n = 40, CI95% 0.6 – 1.6 nM, range 0.2 – 42.2), respectively.

The relationships between increased *pfmdr1 *copy number and in vitro drugs susceptibilities were then analysed. *Pfmdr1 *copy number has been regouped into low (< 2 copies) and high (≥ 2 copies). Parasites with increased *pfmdr1 *copy number demonstrated significantly reduced susceptibilities to mefloquine and lumefantrine (p < 0.01, Table 1). These strains also showed reduced susceptibilities to endoperoxides and increased susceptibility to chloroquine, but these differences did not reach significant levels. No association was found with in vitro susceptibility to quinine (Table 1). Similarly, no association was found between *pfmdr1 *genotype and in vitro results for any tested drugs.

**Table 1:** Correlation between IC_50 _and *pfmdr1 *copy number outcome.

	Single *pfmdr1 *copy number		Increased *pfmdr1 *copy number**		p*
			
	Number	Mean IC_50 _(range) nM/l	Number	Mean IC_50 _(range) nM/l	
Mefloquine	73	27.9 (2.3 – 132.4)	17	50.3 (9.6 – 132.9)	0.007
Artesunate	80	1.6 (0.3 – 11.1)	17	2.1 (0.6 – 5.6)	0.054
Lumefantrine	45	21.4 (3.6 – 63.5)	5	44.1 (25.9 – 79.2)	0.006
Artemether	33	2.2 (0.2 – 17)	7	7.6 (0.2 – 42.2)	0.143
Quinine	73	158.2 (26.1 – 615.9)	16	172.3 (19.9 – 612.2)	0.677
Chloroquine	73	235.4 (54.1 – 663.3)	16	175.6 (71.4 – 331.9)	0.085

*Significance level (p) was 0.05. The analysis was tested by Wilcoxon rank-sum (Mann-Whitney) test.

**The cutoff of *pfmdr1 *multicopy was 2 copies.

### In vivo study

In vivo outcome was reported previously by Denis MB et al, 2006 [8,9] and is summarized in table 2. As determined by *msp1 *, *msp2 *and *glurp *genotyping, 10 in the AM group and 23 in the AL group were classified as recrudescence. All failures were late treatment failures (LTF = LCF + LPF). In summary, there were 96 (81%) ACPR and 23 (19%) LTF in AL group and 123 (92.5%) ACPR and 10 (7.5%) LTF in the AM group (Figure 1).

**Table 2:** In vivo outcome measures

Study	Number of patients			Treatment outcome (n. %)			
		
	Included	Drop out*	Analysed	ACPR	LTF	LPF	LCF
AL2002†	55	10(4)	45	32(71.1)	13(28.9)	2(4.4)	11(24.5)
AL2003†	80	6(3)	74	64(86.5)	10(13.5)	6(8.1)	4(5.4)
AM2003‡	55	3(0)	52	48(92.3)	4(7.7)	3(5.7)	1(2)
AM2004‡	85	4(1)	81	75(92.6)	6(7.4)	0(0)	6(7.4)

ACPR. Adequate Clinical and Parasitological Response; LTF. Late Treatment Failure;

LCF. Late Clinical Failure; LPF. Late Parasitological Failure; LTF = LCF + LPF.

*Number between parenthesis indicates the number *Plasmodium vivax *infections

†Artemether 20 mg/kg and lumefantrine 120 mg/kg

‡Artesunate 12 mg/kg and mefloquine 25 mg/kg

**Figure 2:**
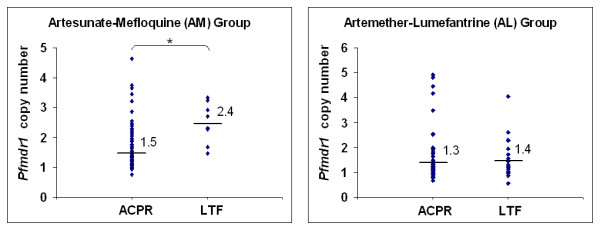
**Comparison of *pfmdr1 *copy number and clinical failure outcome**. Artesunate-mefloquine: ACPR (N = 103, copy number ≥ 2 = 20); LTF (N = 9, copy number ≥ 2 = 7); * p < 0.001. Artemether-lumefantrine: ACPR (N = 76, copy number ≥ 2 = 8); LTF (N = 20, copy number ≥ 2 = 4), p = 0.257 (the analysis was tested by Wilcoxon rank-sum (Mann-Whitney) test). The black horizontal lines and number represent geometric mean *pfmdr1 *copy number.

Pfmdr1 copy number was lower in the ACPR group compared to the LTF group (n = 112, p < 0.001) for AM but not for AL treatment (n = 96, p = 0.257) (Figure 2). The proportional hazards assumption was violated when pfmdr1 copy number was classified as a single copy versus multiple copies. Therefore, to allow statistical analysis of copy number data, two groups were build: low (< 3 copies) and high (≥ 3 copies). A pfmdr1 copy number that is ≥ 2.5 is rounded up to 3. This way of coding copy number met the proportional hazards assumption.

**Figure 3:**
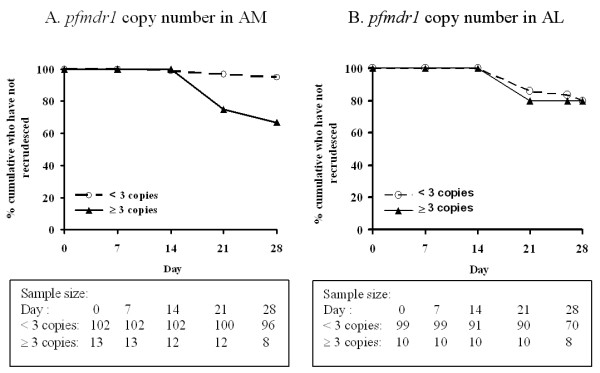
**The relationship between *pfmdr1 *copy number (A, B) and time to recrudescence (the relationship was estimated using survival analysis)**.

In the AM group (N = 115), crude hazard ratio for increased copy number (≥ 3 copies compared to < 3 copies) was 7.80 [95%CI: 2.09–29.10], p = 0.002. After controlling for baseline parasitaemia and gender in a Cox's regression model, the hazard ratio was 12.28 [95%CI: 3.19–47.30], p < 0.001. There was no association between increased copy number and treatment failure in the AL group [HR = 1.03 [95%CI: 0.24–4.46], p = 0.962] (Figure 3).

## Discussion

The involvement of *pfmdr1 *in anti-malarial resistance has been suspected since the 1990's but practical use of this information had to wait for methodological improvements and the availability of high quality quantitative PCR in endemic areas. This is now possible in a basic molecular laboratory. In this study, increased *pfmdr1 *copy number was associated with AM recrudescence. The association between *pfmdr1 *copy number and treatment failure to mefloquine-based regimens has been consistently found in both Cambodia and Thailand [5-7]. In addition, mefloquine monotherapy and combination therapy has previously been shown to select for increased *pfmdr1 *copy number within a host [5,19]. Increased *pfmdr1 *copy number was also associated with decreased in vitro susceptibility to mefloquine, lumefantrine and, to a lesser degree artesunate, which is consistent with previous studies [20-22]. It is not clear whether the association between *pfmdr1 *copy number and AM failure is mediated by resistance to mefloquine alone or in conjunction with decreased sensitivity to artesunate. Further studies of this question are required.

In vitro drug sensitivity was successfully assayed in only 44% of the blood specimens. This high failure rate is likely to result from low parasite counts. However, some other factors such as poor transport condition from field sites to laboratory, or prolonged storage of infected blood at 4°C or presence of drug trace in the sample may have similarly interfere with the performance of the test [4]. These factors raise the possibility that the failure of certain strains to grow in vitro may not have been determined randomly, but may have been associated, for example, with decreased fitness, perhaps linked to decreased drug sensitivity. Therefore, measured in vitro IC_50_s may not be representative of the full set of samples studied for clinical outcomes and molecular features.

Interestingly, *pfmdr1 *copy number had apparently no impact on clinical AL failure even though it was associated with decreased in vitro susceptibility to both artemether and lumefantrine [19,22]. This could be related to the overall low *pfmdr1*-associated increase of IC_50_'s for these drugs (Table 1). Another possibility is that the AL clinical failures in this study might result from poor lumefantrine absorption. Actually, oral bioavailability of lumefantrine has been shown to be considerably reduced during the acute phase of malaria and also to vary from one individual to another. It is therefore possible that the fraction absorbed by patients did not reach optimal curative levels, leaving unaffected some residual parasites. However, this possibility is probably not major issue in this study as an association between increased *pfmdr1 *copy number and decreasing in vitro susceptibility to lumefantrine was at the same time observed, suggesting that AL failure could indeed result from decreased susceptibility to lumefantrine. Because of the exponential growth of Coartem^® ^use in the past 5 years, high selection pressure could also be acting in Africa. The monitoring of drug resistance to lumefantrine is thus important for national anti-malarial treatment policy particularly in African countries.

## Conclusion

This study shows that *pfmdr1 *copy number is a molecular marker of AM treatment failure in *falciparum *malaria on the Cambodia-Thailand border. However, while it is associated with increased IC_50 _for lumefantrine, *pfmdr1 *copy number is not associated with AL treatment failure in the area, suggesting involvement of other factors or molecular mechanisms in AL treatment failures in Cambodia.

## Competing interests

The authors declare that they have no competing interests.

## Authors' contributions

PL did molecular investigation, carried out in vitro susceptibility testing of the field samples and drafted the paper. APA, NKS, and SRM developed the *pfmdr1 *assay. CB and NK sequenced the samples. The clinical studies were coordinated and supervised by SD, MBD and PY. The study of molecular resistance markers was initiated by OMP, TF, SI and CW. FA, PR, JLB conceived the study, participated in study design. FA performed quality control of data and helped to draft the paper. PR is a staff member of the World Health Organization. This author alone is responsible for the views expressed in this publication and they do not necessarily represent the decisions, policy or views of the World Health Organization. All authors contributed to the critical review of the manuscript and agree to submission.

## References

[B1] Eyles DE, Hoo CC, Warren M, Sandosham AA (1963). *Plasmodium falciparum *resistant to chloroquine in Cambodia. Am J Trop Med Hyg.

[B2] WHO (2005). Review of the malaria drug efficacy situation.

[B3] WHO (2002). Development of South-Asia surveillance network for malaria drug resistance. Report on an informal consultative meeting.

[B4] Lim P, Chim P, Sem R, Nemh S, Poravuth Y, Lim C, Seila S, Tsuyuoka R, Denis MB, Socheat D, Thierry F (2005). In vitro monitoring of *Plasmodium falciparum *susceptibility to artesunate, mefloquine, quinine and chloroquine in Cambodia: 2001–2002. Acta Trop.

[B5] Alker AP, Lim P, Sem R, Shah NK, Yi P, Bouth DM, Tsuyuoka R, Maguire JD, Fandeur T, Ariey F, Wongsrichanalai C, Meshnick SR (2007). *Pfmdr1 *and in vivo resistance to artesunate-mefloquine in falciparum malaria on the Cambodian-Thai border. Am J Trop Med Hyg.

[B6] Nelson AL, Purfield A, McDaniel P, Uthaimongkol N, Buathong N, Sriwichai S, Miller RS, Wongsrichanalai C, Meshnick SR (2005). *pfmdr1 *genotyping and in vivo mefloquine resistance on the Thai-Myanmar border. Am J Trop Med Hyg.

[B7] Price RN, Uhlemann AC, Brockman A, McGready R, Ashley E, Phaipun L, Patel R, Laing K, Looareesuwan S, White NJ, Nosten F, Krishna S (2004). Mefloquine resistance in *Plasmodium falciparum *and increased *pfmdr1 *gene copy number. Lancet.

[B8] Denis MB, Tsuyuoka R, Lim P, Lindegardh N, Yi P, Top SN, Socheat D, Fandeur T, Annerberg A, Christophel EM, Ringwald P (2006). Efficacy of artemether-lumefantrine for the treatment of uncomplicated falciparum malaria in northwest Cambodia. Trop Med Int Health.

[B9] Denis MB, Tsuyuoka R, Poravuth Y, Narann TS, Seila S, Lim C, Incardona S, Lim P, Sem R, Socheat D, Christophel EM, Ringwald P (2006). Surveillance of the efficacy of artesunate and mefloquine combination for the treatment of uncomplicated falciparum malaria in Cambodia. Trop Med Int Health.

[B10] WHO (2003). Assessment and monitoring of antimalarial drug efficacy for the treatment of uncomplicated falciparum malaria. (WHO/HTM/RBM/200350).

[B11] Desjardins RE, Canfield CJ, Haynes JD, Chulay JD (1979). Quantitative assessment of antimalarial activity in vitro by a semiautomated microdilution technique. Antimicrob Agents Chemother.

[B12] Sakihama N, Mitamura T, Kaneko A, Horii T, Tanabe K (2001). Long PCR amplification of *Plasmodium falciparum *DNA extracted from filter paper blots. Exp Parasitol.

[B13] Durrand V, Berry A, Sem R, Glaziou P, Beaudou J, Fandeur T (2004). Variations in the sequence and expression of the *Plasmodium falciparum *chloroquine resistance transporter (Pfcrt) and their relationship to chloroquine resistance in vitro. Mol Biochem Parasitol.

[B14] Snounou G, Zhu X, Siripoon N, Jarra W, Thaithong S, Brown KN, Viriyakosol S (1999). Biased distribution of msp1 and msp2 allelic variants in *Plasmodium falciparum *populations in Thailand. Trans R Soc Trop Med Hyg.

[B15] Khim N, Bouchier C, Ekala MT, Incardona S, Lim P, Legrand E, Jambou R, Doung S, Puijalon OM, Fandeur T (2005). Countrywide survey shows very high prevalence of *Plasmodium falciparum *multilocus resistance genotypes in Cambodia. Antimicrob Agents Chemother.

[B16] http://www.malaria.mr4.org.

[B17] Ferreira ID, Rosario VE, Cravo PV (2006). Real-time quantitative PCR with SYBR Green I detection for estimating copy numbers of nine drug resistance candidate genes in *Plasmodium falciparum*. Malar J.

[B18] Wellems TE, Panton LJ, Gluzman IY, do Rosario VE, Gwadz RW, Walker-Jonah A, Krogstad DJ (1990). Chloroquine resistance not linked to mdr-like genes in a *Plasmodium falciparum *cross. Nature.

[B19] Uhlemann AC, McGready R, Ashley EA, Brockman A, Singhasivanon P, Krishna S, White NJ, Nosten F, Price RN (2007). Intrahost selection of *Plasmodium falciparum pfmdr1 *alleles after antimalarial treatment on the northwestern border of Thailand. J Infect Dis.

[B20] Pickard AL, Wongsrichanalai C, Purfield A, Kamwendo D, Emery K, Zalewski C, Kawamoto F, Miller RS, Meshnick SR (2003). Resistance to antimalarials in Southeast Asia and genetic polymorphisms in *pfmdr1*. Antimicrob Agents Chemother.

[B21] Price RN, Cassar C, Brockman A, Duraisingh M, van Vugt M, White NJ, Nosten F, Krishna S (1999). The *pfmdr1 *gene is associated with a multidrug-resistant phenotype in *Plasmodium falciparum *from the western border of Thailand. Antimicrob Agents Chemother.

[B22] Price RN, Uhlemann AC, van Vugt M, Brockman A, Hutagalung R, Nair S, Nash D, Singhasivanon P, Anderson TJ, Krishna S, White NJ, Nosten F (2006). Molecular and pharmacological determinants of the therapeutic response to artemether-lumefantrine in multidrug-resistant *Plasmodium falciparum *malaria. Clin Infect Dis.

